# Experimental investigation of exercise-related hedonic responses to preferred and imposed media content

**DOI:** 10.15171/hpp.2018.14

**Published:** 2018-04-18

**Authors:** Emily Frith, Paul D. Loprinzi

**Affiliations:** ^1^Exercise Psychology Laboratory, Physical Activity Epidemiology Laboratory, Department of Health, Exercise Science and Recreation Management, The University of Mississippi, University, MS 38677, USA

**Keywords:** Health promotion, Motivation, Physical activity, Social media

## Abstract

**Background:** We evaluated the differential influence of preferred versus imposed media selections on distinct hedonic responses to an acute bout of treadmill walking.

**Methods:** Twenty university students were recruited for this [160 person-visit] laboratory experiment, which employed a within-subject, counter-balanced design. Participants were exposed to 8 experimental conditions, including (1) Exercise Only, (2) Texting Only, (3) Preferred Phone Call, (4) Imposed Phone Call, (5) Preferred Music Playlist, (6) Imposed Music Playlist, (7)Preferred Video and (8) Imposed Video. During each visit (except Texting Only), participants completed a 10-minute bout of walking on the treadmill at a self-selected pace. Walking speed was identical for all experimental conditions. Before, at the midpoint of exercise, and post-exercise, participants completed the Feeling Scale (FS) and the Felt Arousal Scale (FAS) to measure acute hedonic response. The Affective Circumplex Scale was administered pre-exercise and post-exercise.

**Results:** Significant pre-post change scores were observed for happy (Imposed Call: P=0.05;Preferred Music: P=0.02; Imposed Video: P=0.03), excited (Exercise Only: P=0.001; PreferredVideo: P=0.01; Imposed Video: P=0.03), sad (Preferred Music: P=0.05), anxious (ExerciseOnly: P=0.05; Preferred Video: P=0.01), and fatigue (Exercise Only: P=0.03; Imposed Video:P=0.002). For the FS all change scores were statistically significant from pre-to-mid and pre-topost (P<0.05).

**Conclusion:** This experiment provides strong evidence that entertaining media platforms substantively influences hedonic responses to exercise. Implications of these findings are discussed.

## Introduction

A digital revolution is dominating the modern fitness industry. Smart phones offer portable, personal, and customizable features, which interact to offer individuals the opportunity to mix physical activity and entertainment into a heterogeneous cocktail of unprecedented social autonomy. Most modern gym spaces feature cardio machines equipped with iPod docks, interactive display screens simulating outdoor environments, and constant television access. Further, exercise facilities continuously broadcast popular music from public access systems, so even patrons exercising without their digital devices will be subjected to imposed media for the duration of their gym activities.^1^ The popularity of wearable fitness tracking applications (apps) has increased in free-living physical activity settings,^2^ and modern music applications have developed algorithms to build personalized playlists for individual subscribers.^3^ Health and wellness apps are cost-effective and enable viral dissemination of information,^4^ thus promoting the transcendence of social support networks spanning race, social class, education, and economic status. Although, despite revolutionary advances in contemporary fitness technology, the use of mobile devices is correlated with lack of physical activity and fitness level among college students^5^ The omnipresent use of modern technology is especially pronounced in the young adult population, with self-reported time spent using mobile devices more than doubling the recommended 150 minutes of weekly moderate-to-vigorous (MVPA) physical activity.^6,7^ To date, there is a lack of research examining the use of multimedia as a tool to aid physical activity adoption, and not as leisure time entertainment.^6^

It is important to specifically evaluate college students under the lens of both media consumption and exercise, as university attendees are susceptible to incremental weight gain, which may be partially due to time constraints and decreased physical activity participation throughout the college years.^6,8^ College-aged individuals have also been described as the most digitally active subset of the global population, with many millennials sleeping with their Smart Phones.^9,10^ essentially treating social media as an inseparable extension of the self. Theoretically, mobile devices may serve as an advantageous diversion of attention from effortful exercise,^11^ emotionally modulating perceptions of exertional fatigue (mechanistically referred to as “attentional processing”). Certain media platforms may also potentiate subliminal motivational cues for exercise continuation,^12^ perhaps enhancing the possibility for enjoyable physical activity adherence and long-term health behavior change. Theories of implicit motivation underscore the importance of delineating both quality and context associated with presentation of media content, as well as personal interpretations of social media stimuli. Nevertheless, an additive impact of distraction during physical activity, emotional response from the media content, and enhanced motivation could perhaps augment enjoyment of physical activity, arousal control, and mitigation of perceived exertion.^13^ Thus, the purpose of the present study was to examine the differential influence of preferred versus imposed media selections on distinct theory-based, hedonic responses to an acute bout of treadmill walking, which was selected as walking is the most common modality of exercise among adults.^14^

Research investigating music exposure during exercise has indicated that dissociation of exercise-oriented thought processes is mediated by feelings linked with song characteristics.^15^ Additionally, the use of music may impede interceptive afferent negative stimuli from communicating with sensory regions of the brain.^15,16^ The possibility for alternative forms of multimedia to exert similar dissociative effects on emotional, rather than cognitive processing is less investigated. However, previous work has shown video content may provide a distraction sufficient to increase time spent exercising, but concomitantly reduce the drive to increase intensity across the exercise bout.^11^ In fact, in Rebold and colleagues’ seminal publication investigating the effects of varied multimedia scenarios on individual heart rate response, speed selection, and liking of a 30-minute bout of treadmill exercise, only music was shown to favorably influence intensity.^17^ The researchers utilized 2 additional conditions of media interaction, texting and talking, for this experiment. However, researcher-selected impositions were shown to induce an interference effect on participant-selected workload. The authors propose this distraction could be problematic when individuals are attempting to improve cardiorespiratory fitness. Nevertheless, talking on the phone was shown to enrich the subjective exercise experience within subjects.^17^ Couched within a media framework and hedonic theory underpinnings, the present study aims to capitalize upon the importance of these findings, by further evaluating the link between use of digital media and emotional reactivity to exercise. Much like Rebold’s research, we utilized a within-subjects experimental design, and manipulated texting, talking on the phone, and listening to music. However, we additionally examined affectual responses to video exposure. The four media scenarios were counterbalanced across 8 visits. It is worthwhile to differentiate affective outcomes between participant-selected versus researcher-imposed conditions, especially as Rebold’s self-selected music group not only experienced significant experimental results, but was also the only condition permitting media self-selection. Although external media may be a distracting stimulus, it may also provide substantial aesthetic improvements within the environmental context.^18^ To this end, the implications of social media entertainment, evaluated under the lens of health promotion research, may encourage exercise enjoyment and, potentially, future adherence to habitual physical activity participation.

## Materials and Methods

### Study design and participants

**Figure 1:**
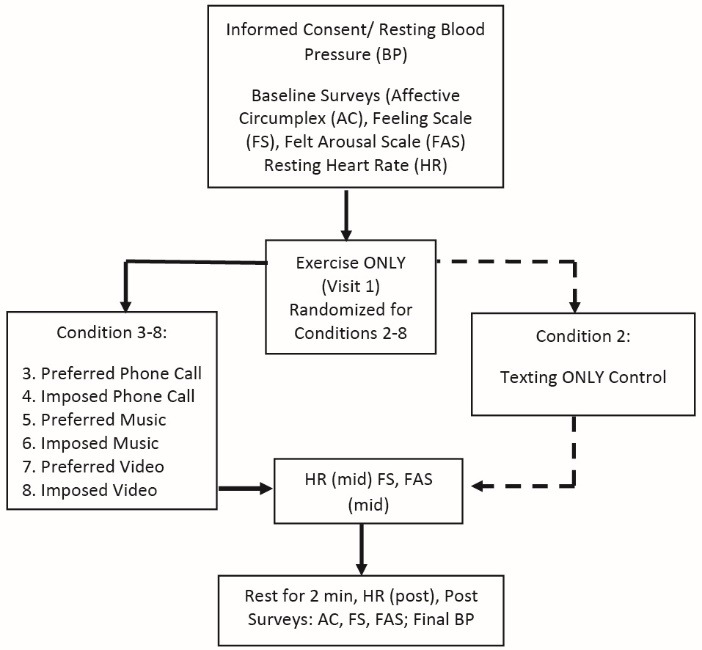
Schematic of the study procedures.

This experiment was conducted utilizing a within-subject, counter-balanced design. See Figure 1 for a schematic of the study procedures. The study was comprised of 8 experimental visits to the laboratory, over a four-week period. Each participant came to the laboratory bi-weekly, with at least 24 hours between visits. We recruited 22 participants via a convenience-based sampling approach. Two individuals were excluded at baseline after meeting exclusionary criteria; thus, 20 participants comprised the analytic sample, which included 160 total visits to the laboratory (20 participants x 8 experimental visits). The sample size for this within-subject experimental design is similar to other related studies.^19-22^ The specific exclusionary questions are detailed below.

*“Are you a smoker? Are you currently taking medication for epilepsy, depression, or anxiety? What is your average alcohol consumption? (“1 drink is defined as a 12-oz. beer, 4 oz. glass of wine, or 1 oz. of liquor.”) Have you used marijuana or other illegal drugs within the past 30 days?*

*Please rate your like/dislike for exercising on a treadmill, based on your agreement with this statement, “I dislike exercising on a treadmill” (1 to 10 Likert-scale) 1 = strongly disagree; 10 = strongly agree.”*

An individual was excluded if he or she answered “Yes” to current smoker status, medication known to alter mood or mental state, drinking an excess of 7 alcoholic beverages per week for females or more than 14 per week for males. If an individual rated their subjective treadmill dislike as greater than 7, they were excluded from participation. This was considered because baseline aversion to treadmill exercise may have moderated participants’ global affect, irrespective of experimental condition. All subjects meeting inclusionary criteria agreed to participate for the remainder of the study. Thus, we lost no participants to attrition.

### Experimental conditions

There were 8 counterbalanced, experimental conditions, which included (1) Exercise Only, (2) Texting Only, (3) Preferred Phone Call, (4) Imposed Phone Call, (5) Preferred Music Playlist, (6) Imposed Music Playlist, (7) Preferred Video and (8) Imposed Video. Details of these experimental conditions are described in the narrative that follows. As stated above, the experimental conditions occurred in a randomized order. However, for all participants, the first visit to the laboratory was the Exercise Only (Condition 1) condition. During this visit, participants completed a 10-minute bout of walking on the treadmill at a self-selected pace (described below). For each of the subsequent visits, they walked on the treadmill for 10 minutes at the pace they self-selected during Condition 1; thus, walking speed was identical for all experimental conditions. The conditions (e.g., texting only, phone call, music, video, etc.) for Visit 2 through Visit 8 occurred in a randomized order across the participants.

### Condition 1- Exercise Only 

Each participant arrived at our lab for their baseline orientation visit. Upon signed authorization of the informed consent document, the lead investigator read the Physical Activity Readiness Questionnaire (PAR-Q) to the participant. If no emergent contraindications presented, every participant was asked to self-report their age, gender, and race-ethnicity as baseline demographic information. Participants were also asked questions to ensure they met predetermined inclusionary criteria. After meeting inclusionary criteria, current physical activity habits were assessed using the Physical Activity Vital Sign assessment (PAVS). The PAVS is a brief survey tool, composed of 2 questions. (1) “How many days per week, on average do you engage in moderate to vigorous intensity physical activity (including a brisk walk),” and (2) “How many minutes, on average do you engage in this physical activity?” This simple questionnaire has been shown to exhibit adequate reliability,^23^ and is correlated with accelerometer-assessed number of days 30 bout-min MVPA (*r* = 0.52, *P* < 0.001).^24^

### General protocol for all experimental conditions

Heart rate was monitored continuously throughout each visit using a chest-mounted Polar HR monitor. After first putting on the heart-rate monitor, participants were given several surveys to complete. Participants completed surveys in a quiet laboratory space, free from distraction. The surveys, in this order, included the Affective Circumplex Scale, the Feeling Scale (FS), and the Felt Arousal Scale (FAS), which are detailed as follows. These affect assessments were chosen because of their straightforward assessment, evidence of construct validity, and ability to differentiate basic and distinct affect.^25,26^

*The Affective Circumplex (8 items)*

This scale is based off of the orthogonal Affective Circumplex Model describing valence and emotional status. The researcher asked the participant to make a tic mark along an 11 mm (0-100) 2-dimensional scale of present emotional state. Marks closer to 0 indicated disagreement with the survey item, while marks closer to 100 indicated agreement with the item, as a representation of their affect in the present moment. Sample items include *‘Happy, Content, Sad, and Fatigued.’*

*Feeling Measure Scale*

The FS was administered to the participants to assess how they felt in the present moment. As feelings are principally transient emotions, that are subject to change numerous times throughout the day. The FS was an appropriate measure to use as an index of valence for the present experiment. The participant was asked to circle the number with which they agreed with the most at this moment in time. The scale ranges from -5 to +5, and every other integer is described as follows: *‘+5 = very good, +3 = good, + 1 = fairly good, 0 = neutral, - 1 = fairly bad, -3 = bad, and -5 = very bad.’* Original validation of the FS for use during exercise has been shown by Hardy and Rejeski.^25^

*Felt Arousal Scale*

The FAS was administered to the participants to assess their perceived arousal in the present moment.^26^ The FAS assesses arousal level, which is readily modulated by stimuli in the external and internal environment. The participants were asked to circle the number with which they most agreed with in the present moment. The scale ranges from 1 to 6, with verbal anchors denoting 1 as corresponding with low arousal and 6 with high arousal. Per the FAS, high arousal is defined as parameters such as *excitement, anxiety, or anger*. Low arousal is characterized as parameters such as *relaxation, boredom, or calmness*.

The scales used in this study to assess affect and arousal have demonstrated evidence of convergent validity and sensitivity to change in numerous investigations.^27,28^ Thus, the measures selected were appropriate to assess our identified outcomes pertaining to hedonic theory and emotional reactivity during exercise.

After the participants were finished completing these scales, a resting heart rate was recorded and the participant was instructed to, *“Please walk on the treadmill at a pace you would be comfortable talking on the phone, listening to music, or watching video content. Your breathing should not be labored. This is going to be a leisurely pace you could enjoy for 10 minutes. You will be asked to walk at this same speed for each visit throughout the study.”* Participants were asked to adjust the treadmill to a stable, comfortable speed within the first minute. Participants were also assured that the researcher would leave the laboratory during the exercise bout, and only enter briefly at the midpoint (5 minutes) of the 10-minute walking session, and during the final minute, to record heart rate and rating of perceived exertion, which ranges from a minimal rating of 6, or no exertion, to 20, or maximal exertion (Borg Rating of Perceived Exertion). The participant was also informed that they would be asked to complete 2 brief scales (FS and FAS), secured to a clipboard, at the midpoint. After participants responded, they would present the clipboard to the researcher facing down, so the researcher would be blinded to the participants’ response.

Immediately after a final heart rate and RPE were recorded, and participants had walked for 10 minutes, the same three scales (Affective Circumplex, FS, and FAS) were administered to participants, and the researcher again stepped out of the laboratory space to give participants adequate time and privacy to respond honestly on all items. After participants were finished, the researcher entered the lab to record an end-of-visit heart rate, and explained that participants were to email or text the researcher three songs, one YouTube video (at least 10 minutes in length), and identify a friend or family member who would be available to talk on the phone for 10 minutes during the participants’ next visit. Visits were scheduled with the participant to occur bi-weekly for four weeks, and both the researcher and participant were blinded to the randomization condition the participant would be exposed to during the subsequent visits. Each visit followed the general protocol described for Condition 1, with only the exercise bout manipulated for differential media exposure.

Before leaving the laboratory, height (cm) and weight (kg) were recorded. Height was measured at baseline only, while weight was measured once per week.

### Condition 2- Texting Only

Participants arrived at the lab with music, video, or an available phone contact prepared per intervention instructions. A resting heart rate was recorded, and participants were asked to “*Please sit quietly for 10 minutes while texting on your phones. Please try to avoid checking social media, and just text. I will not ask you who you are texting, or to view any of your texts.”* Participants were also asked how many people they texted during their 10-minute session, and this value was recorded.

### Condition 3- Preferred Call

Participants arrived at the lab with music, video, or an available phone contact prepared per intervention instructions. Participants were told they had been randomized into the preferred call condition. Participants were asked to *“Please walk on the treadmill at the exact same speed you selected during your first visit. Please start walking when the person you call picks up the phone. At that point, I will leave the laboratory for you to enjoy your conversation. However, I will still enter at the midpoint, and during the final minute of your walk.”* The researcher told participants to stop the treadmill at 10-minutes, but allowed two minutes for the participants to end their phone calls respectfully. After participants were finished, the researcher entered the lab to record an end-of-visit heart rate, and ask the relationship the participant had to the chosen caller (i.e. friend or family).

### Condition 4- Imposed Call

Participants arrived at the lab with music, video, or an available phone contact prepared per intervention instructions. Participants were told they had been randomized into the preferred call condition. Participants were given the following instructions: *“I am going to leave the laboratory to find a quiet place to call you. Please start walking when the you answer my phone call. Make sure your phone is placed in one of the cup-holders, and that you can hear me clearly through the headphones. Also, please walk on the treadmill at the exact same speed you selected during your first visit. I will not enter at the midpoint, or during the final minute of your walk. The scales are on a clipboard on a stool I have placed next to the treadmill. I will tell you to straddle the treadmill belt, and fill them both out at the five-minute mark of our phone call.”* After the phone call began, the researcher used the following preliminary script to initiate conversation: *“I have heard that, in life, it is important to focus your energy on three main things. The first is your livelihood, or your professional ambitions; the second is your health. This could be physical health, mental health, spiritual health, or some combination; and the third area is your personal creativity. I’m going to share a bit about myself with respect to each of these, and ask you to share your thoughts as well.”* The conversation was permitted to flow naturally after this point, as the prompt was chosen to facilitate enjoyable conversation, despite speaking with a stranger. If the topics were exhausted, and there was still time remaining, the researcher would ask *“Who has been the most influential person in getting you to where you are today and why?* The researcher asked participants to verbalize their heart rate and RPE at the midpoint of the imposed phone conversation. Researchers asked participants to verbalize their heart rate and RPE during the last minute of exercise. The researcher told participants to stop the treadmill at 10-minutes, ended the call, and entered the lab shortly thereafter.

### Condition 5- Preferred Music

Participants arrived at the lab with music, video, or an available phone contact prepared per intervention instructions. Participants were told they had been randomized into their preferred music condition. Bluetooth headphones were paired with a Lenovo Yoga iPAD, on which the participant’s three chosen songs had been added to an individualized playlist the researcher created using the free music application Hypster. Participants were asked to *“Please walk on the treadmill at the exact same speed you selected during their first visit. Please start walking when you can hear the music playing through the headphones. Let me know if I need to adjust the volume.”* The researcher placed the iPad on the treadmill facing down, so the participant would not see any videos or images associated with the songs, ensuring exclusive exposure to audio. The 6-item Brunel Music Rating Inventory Form-B^12^ was administered along with the previously utilized post-exercise scales. This survey assesses the importance of distinct motivational qualities of music on a 7-point Likert-scale, with higher numeric ratings corresponding to higher motivational quality.

### Condition 6: Imposed Music 

Participants arrived at the lab with music, video, or an available phone contact prepared per intervention instructions. Participants were told they had been randomized into the imposed music condition. Bluetooth headphones were paired with a Lenovo Yoga iPAD, on which the researcher’s three chosen songs from the US Billboard Hot 100 Songs of 2016, and screened for no negative emotions (Linguistic Inquiry and Word Count analysis) had been added to a playlist the researcher created using the free music application Hypster. Participants were asked to *“Please walk on the treadmill at the exact same speed you selected during their first visit. Please start walking when you can hear the music playing through the headphones. Let me know if I need to adjust the volume.”* The researcher placed the iPad on the treadmill facing down, so the participant would not see any videos or images associated with the songs, ensuring exclusive exposure to audio. The 13-item Brunel Music Rating Inventory Form-A,^12^ was administered along with the previously utilized post-exercise scales. This survey assesses the importance of distinct motivational qualities of music on a 10-point Likert-scale, with higher numeric ratings corresponding to higher motivational quality.

### Condition 7- Preferred Video

Participants arrived at the lab with music, video, or an available phone contact prepared per intervention instructions. Participants were told they had been randomized into their preferred video condition. Bluetooth headphones were paired with a Lenovo Yoga iPAD, on which the participant’s video was loaded on YouTube. Participants were asked to *“Please walk on the treadmill at the exact same speed you selected during their first visit. Please start walking when you can hear the video playing through the headphones. Let me know if I need to adjust the volume.”* The researcher placed the iPad on the treadmill facing up, so the participant could clearly see the video content or images associated with the songs.

### Condition 8- Imposed Video

Participants arrived at the lab with music, video, or an available phone contact prepared per intervention instructions. Participants were told they had been randomized into the imposed video condition. Bluetooth headphones were paired with a Lenovo Yoga iPAD, on which the researcher’s video (Lip Sync Battle with Will Ferrell, Kevin Hart and Jimmy Fallon, length-13:12) was loaded on YouTube. Participants were asked to *“Please walk on the treadmill at the exact same speed you selected during their first visit. Please start walking when you can hear the video playing through the headphones. Let me know if I need to adjust the volume.”* The researcher placed the iPad on the treadmill facing up, so the participant could clearly see the video content or images associated with the songs.

### Statistical Analysis

**Figure 2:**
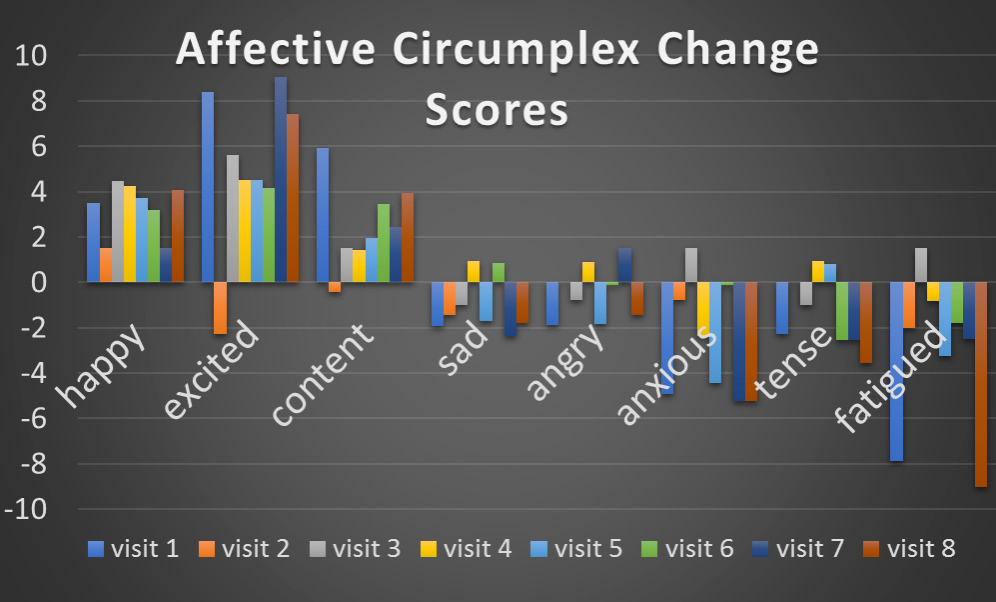
Mean affective circumplex change scores across the 8 visits. Each individual change score represents the post-exercise minus pre-exercise affect change score for each distinct affect parameter across each respective visit. With regard to the significant pre-post change scores for the individual visits, statistical significance was observed for happy (visit 4: *P* = 0.05; visit 5: *P* = 0.02; visit 8: *P* = 0.03), excited (visit 1: *P* = 0.001; visit 7: *P* = 0.01; visit 8 *P* = 0.03), sad (visit 5: *P* = 0.05), anxious (visit 1: P=0.05; visit 7: *P* = 0.01), and fatigued (visit 1: *P* = 0.03; visit 8: *P* = 0.002). Condition description: visit 1-Exercise Only; visit 2-Texting Only; visit 3-Preferred Call; visit 4- Imposed Call; visit 5-Preferred Music; visit 6-Imposed Music; visit 7-Preferred Video; visit 8-Imposed Video.

**Figure 3:**
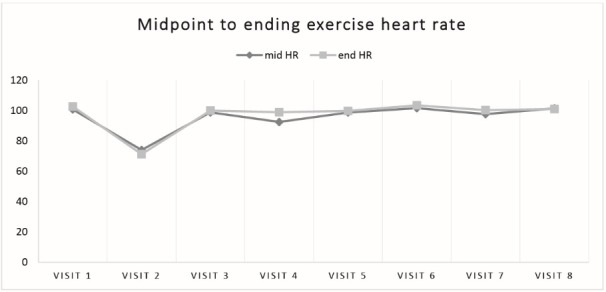
Values for heart rate were recorded during exercise across the 8 visits. Each individual data point represents the mean recorded heart rate for the midpoint of the treadmill walk (dark gray line) and endpoint of the treadmill walk (light gray line). Condition description: visit 1-Exercise Only; visit 2-Texting Only; visit 3-Preferred Call; visit 4- Imposed Call; visit 5-Preferred Music; visit 6-Imposed Music; visit 7-Preferred Video; visit 8-Imposed Video.

Stata SE Version 12 (College Station, Texas, USA) was used to calculate univariate results shown in Table 1. We also generated change scores for the Affective Circumplex, FS, and FAS measures. For these measures, change scores were calculated for pre-to-mid, mid-to-post, and pre-post. We used these change scores to conduct paired-samples t-tests to examine the differences in change scores for the Affective Circumplex (Figure 1), FS (Table 2), and FAS (Table 3) across the 8 conditions. The same procedure was used to compute heart rate (Figure 2) and RPE (Figure 3) change scores using paired-samples *t* tests to examine the differences in heart rate and RPE from the midpoint to the end of the exercise bout.

We employed a 2 (time; pre/post) x 8 (conditions) repeated measures analysis of variance (ANOVA) to identify whether a group x time interaction effect was observed (using SPSS v.23). Following this, we computed a one-way repeated measures ANOVA for mean change scores between each visit regarding pre-to-mid and pre-to-post FS, FAS, and Affective Circumplex (pre-post only). For all analyses, statistical significance was established as a nominal alpha of 0.05. The multiple analytic assessments increase the likelihood of committing a type I research error. However, we decided not to correct for multiple comparisons, as the number of type I errors cannot decrease without increasing the risk of making a type II error.^29,30^ Further, the theoretical assumption behind correction for multiple testing is that all null hypotheses are true simultaneously,^29^ which was not of interest in our study.

## Results

### Demographic characteristics 

Study demographics are shown in Table 1, and are reported by a point estimate, standard deviation, and range. The majority (65%) of participants were white. Mean values were computed for variables including age, body mass index (BMI), treadmill speed, attitude towards walking on a treadmill, gender, and positive or negative emotions present in participant- and researcher-chosen music. Negative emotions were not reported for the imposed music playlist, as the linguistic inquiry and word count (LIWC) was used to compute scores for positive and negative emotions. Only songs with a score of 0 for negative emotions were selected for the study. There was a wide range of variability in self-reported MVPA as well as the average self-reported motivation on the Brunel Music Rating Inventory-2 following listening to the researcher-selected playlist.

### Distinct Affect

Mean Affective Circumplex change scores were computed across the 8 visits. Each individual change score shown in Figure 2 represents the post-exercise minus pre-exercise affect change score for each distinct affect parameter across each respective visit. For each distinct affect parameter (e.g., happy), a repeated measures ANOVA (1 x 8) was computed. For the 8 distinct affect parameters, there were no statistically significant (all *P*’s >0.05) trends across all visits, as determined from separate 1 x 8 repeated measures ANOVAs. With regard to the significant pre-post change scores for the individual visits, this was observed for happy (visit 4: *P* = 0.05; visit 5: *P* = 0.02; visit 8: *P* = 0.03), excited (visit 1: *P* = 0.001; visit 7: *P* = 0.01; visit 8: *P* = 0.03), sad (visit 5: *P* = 0.05), anxious (visit 1: *P* = 0.05; visit 7: *P* = 0.01), and fatigued (visit 1: *P* = 0.03; visit 8: *P* = 0.002).

### Valence

From a 2 (time; pre/post) x 8 (conditions) repeated measures ANOVA, there was evidence of an interaction effect (F = 8.6; *P* = 0.008). Paired *t* tests were used to examine differences in mean scores for the feeling scale (possible range of -5 to +5) during each visit pre-exercise, mid-exercise, and immediately following exercise. Pre-to-mid change scores were generated by subtracting the pre-feeling scale rating from the mid-feeling scale rating for each of the 8 visits. As shown in Table 2, *t* tests showed all change scores from pre-to-mid were statistically significant (*P* < 0.05), except visit 2, which was the texting only condition (which served as our control group). No change scores were significant from mid-to-post after subtracting the mid-feeling scale rating from the post-feeling scale rating. All change scores were statistically significant from pre-to-post after computing the difference between the post-feeling scale rating and the pre-feeling scale rating.

SPSS v.23 was used to run a one-way repeated measures ANOVA, which was statically significant for mean change scores between each visit regarding pre-to-mid Feeling Scale along a linear trend (*P* = 0.002). That is, the trend from 0.35 (visit 1) to 0.85 (visit 8) was statistically significant (*P* = 0.002). A one-way repeated measures ANOVA for mean change scores between each visit pre-to-post FS was also statistically significant along a linear trend (*P* = 0.008). That is, the trend from -0.35 (visit 1) to 0.95 (visit 8) was statistically significant (*P* = 0.002).

### Arousal

Paired t-tests were used to compute mean scores for the Arousal Scale (possible range of 1 to 6) during each visit pre-exercise, mid-exercise, and immediately following exercise. Pre-to-mid change scores were generated by subtracting the pre-Arousal Scale rating from the mid-arousal scale rating for each of the 8 visits. As shown in Table 3, t-tests showed all change scores from pre-to-mid were statistically significant (*P < *0.05) for visits 4, 5, 6, 7, and 8. No change scores were significant from mid-to-post after subtracting the mid-Arousal Scale rating from the post-Arousal Scale rating. All change scores except conditions 2 and 3 (texting only, and preferred phone call) were statistically significant from pre-to-post after computing the difference between the post-Arousal Scale rating and the pre-Arousal Scale rating.

SPSS v.23 was used to run a one-way repeated measures ANOVA, which was statically significant for mean change scores between each visit regarding pre-to-mid Arousal Scale along a linear trend (*P* = 0.002). That is, the trend from 0.35 (visit 1) to 0.8 (visit 8) was statistically significant (*P* = 0.009). A one-way repeated measures ANOVA for mean change scores between each visit pre-to-post Arousal Scale was statistically significant along a linear trend (*P* = 0.05). That is, the trend from 0.1 (visit 1) to 0.75 (visit 8) was statistically significant (*P* = 0.05).

### Heart rate and ratings of perceived exertion

Values for heart rate were recorded during exercise across the 8 visits. There were no statistically significant differences for within-subjects effects (*P* = 0.18 and *P* = 0.60 of mid HR and end HR, respectively, from a 1 x 7 repeated measures ANOVA) in average heart rate at the midpoint (5 minutes) or end of exercise (9:45-10 minutes) across the 7 visits that required participants to walk on a treadmill for 10 minutes (Figure 3). Average mid-exercise heart rate for conditions 1 and 3-8 ranged from 92.5 beats per minute to 101.6 beats per minute. Average end-exercise heart rate ranged from 98.9 beats per minute to 99.4 beats per minute for conditions 1 and 3-8. The range of average heart rates for condition 2, which required participants to sit for 10 minutes was 74.0 beats per minute to 71.2 beats per minute.

**Figure 4:**
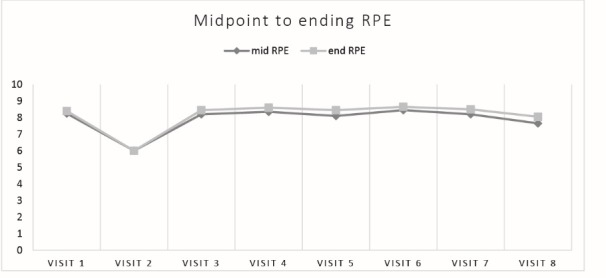
Mean value for subjective rating of perceived exertion (RPE BORG scale) during exercise across the 8 visits. Each individual data point represents the mid-exercise mean reported rating of perceived exertion (dark gray line) and end-exercise mean reported rating of perceived exertion (light gray line). Condition description: visit 1-Exercise Only; visit 2-Texting Only; visit 3-Preferred Call; visit 4- Imposed Call; visit 5-Preferred Music; visit 6-Imposed Music; visit 7-Preferred Video; visit 8-Imposed Video.

Ratings of Perceived Exertion were recorded during exercise across the 8 visits; see Figure 4. There were no statistically significant differences for within-subjects effects (*P* = 0.17 and *P* = 0.50) of mid RPE and end RPE, respectively, from a repeated measures ANOVA) in average RPE at the midpoint (5 minutes) or end of exercise (9:45-10 minutes) across the 7 visits that required participants to walk on a treadmill for 10 minutes. Average mid-exercise RPE for conditions 1 and 3-8 ranged from a low mean of 8.1 (during the preferred music condition) to the highest observed mean of 8.5 (during the imposed music condition). Average end-exercise RPE for conditions 1 and 3-8 ranged from a low mean of 8.1 (imposed video condition) to 8.7 (imposed music condition). During visit 2, the average RPE was 6.

## Discussion

The purpose of this study was to examine the differential influence of preferred versus imposed media selections on distinct hedonic responses to an acute bout of treadmill walking. Each participant visited the laboratory 8 times to participate in a variety of counterbalanced media conditions. We determined the inclusion of two positive control conditions (Exercise Only and Texting Only) was necessary to assess whether affect, feeling, and arousal level were affected differentially between these conditions. We observed no statistical significance in modulation of affect, feeling, or arousal for the social media control, suggesting that exercise alone may exert profound influences on affect, specifically increased excitement and reduced anxiety and fatigue, as well as enhanced feeling and arousal across the exercise bout. Therefore, exercise in isolation may be capable of boosting mood and attenuating negative emotional response, despite social media appearing to have no detrimental impact on emotional appraisal.

The Preferred Call and Imposed Phone Call conditions were both statistically significant in increasing FS scores during the visit. However, arousal was elevated only during the Imposed Call condition, and was more significantly amplified in this condition than in all other visits. Heightened arousal was not an unprecedented finding, as many participants did not know the researcher well, which could have prompted nervousness or unease. Conversely, it is plausible that higher arousal may have been associated with increased enjoyment during the call. In addition to higher positive feelings, participants also rated themselves as happier on the Affective Circumplex measure following the Imposed Call, but not the Preferred Call. One conjectural explanation could be the general framework of the guided conversation. The principal investigator decided to utilize specific interaction prompts, rather than a script of questions, with the intention to create opportunities for deeper conversation. Further, the researcher provided insight into their personally relevant experiences to drive reciprocal engagement and encourage openness and curiosity within an atypical social interaction.^31^ The enhancement of subjective feeling state and acute happiness, respective to the imposed phone call is a novel outcome that should be evaluated in future research. Future research could integrate motivational interviewing, or superior communication strategies, as trivial social interactions between weak ties may be an underexplored mechanism underlying happiness.^32^

Participants’ ratings of feeling state and arousal significantly increased from pre-to-mid exercise, and from pre-to-post exercise in both the Preferred Music versus Imposed Music selections. LIWC software was used to screen popular songs for proportion of negative emotions. Psychometric analysis determined the three chosen songs contained no lyrical references to negative emotions,^33^ which could have potentially impacted feeling and arousal during walking. The range of positive emotions varied widely for participant-selected songs, however the average proportion of positive lyrics in participant- versus researcher-selected music was not statistically significant. Although, despite congruence in positive lyrical content, participants reported significant improvements in happiness, and concomitant reductions in sadness on the Affective Circumplex only for the Preferred Music condition. Additionally, average RPE was lowest during the Preferred Music condition, and highest during the imposed music condition. Therefore, it is plausible that autonomy in participant-selected entertainment may support acute exercise enjoyment.

Average FS improvements were highest during the Preferred Video and Imposed Video conditions when compared to all other conditions. Each condition also significantly increased arousal. Participants reported happiness and excitement, and reduced fatigue following the researcher-selected video. Future research should evaluate diverse video content on emotional reactivity. The video chosen for this study was intended to induce amusement, however alternate selections of video-entertainment could be similarly effective in modulating affective outcomes. Our results also showed that, on average, the participant-selected video increased excitement and reduced anxiety throughout the visit. Future investigations evaluating the practicality of watching videos while exercising is warranted, as this is an underexplored area in health promotion research, and our findings indicate this medium may improve hedonic responses while exercising.

To our knowledge, no study has been conducted investigating the differential effects of various media conditions on a 10-min bout of treadmill walking. Thus, the present study is the most comprehensive experimental investigation evaluating the effects of social media stimuli on hedonic responses to exercise. A limitation of this study was the inability to coordinate schedules to ensure participant visits occurred at exactly the same time of day across all 8 conditions; however, the majority of the conditions occurred within 2 hours of the same time each day. Another limitation is the relatively small sample size; however, we observed numerous statistically significant findings, suggesting that statistical power was not in an issue in this study. Our inclusion of many active individuals may have limited this study’s generalizability to the broader US college-aged population. Active adults are more likely to have developed some degree of intrinsic motivation to exercise. Thus, subsequent research should evaluate the effects of social media while exercising within inactive populations. Nevertheless, this investigation helps advance the field of modern health promotion by highlighting the possibility for entertaining media platforms to influence physical activity enjoyment.

In conclusion, Affective Circumplex ratings, such as happy, excited, sad, anxious, and fatigued, were improved across several of the experimental conditions. Average valence improvements were highest during the Preferred Video and felt arousal was highest during the Imposed Video conditions in comparison to all other conditions. These results underscore the need for effective health promotion efforts to advance in line with modern technology. For example, it would be useful to assess the effects of video content on hedonic responses. Instead of selecting videos intended to induce entertainment or amusement, perhaps researcher-selected video content could incorporate health messages, and/or embed motivational qualities. In addition to selecting videos that have the potential to motivate behavior change, videos could feature music containing a higher proportion of positive lyrics. Subsequent research should also continue to investigate the effects of preferred versus imposed music selection, as this can have significant implications in modern society. The music of contemporary popular culture is not only perpetually imposed in gym and recreational settings, but is also a cost-effective and widely accessible motivational tool for individuals to tailor to their preferences. Although less controllable, social media and physical activity experiments should be conducted in free-living environments. This will allow researchers to examine the plausibility for preferred and/or imposed media content to impact hedonic responses within participant-selected exercise settings.

## Ethical approval

All procedures performed in studies involving human participants were in accordance with the ethical standards of the institutional and/or national research committee and with the 1964 Helsinki Declaration and its later amendments or comparable ethical standards. This study was approved by the authors’ institutional review board (approved protocol No. 17-034) and participant consent was provided before any data collection activities.

## Competing interests

The authors declare that they have no competing interests.

## Authors’ contributions

EF and PL performed analyses. EF collected the data and drafted the manuscript. All authors have reviewed and edited the manuscript. All authors have read and approved the final version of the manuscript, and agree with the order of presentation of the authors.

**Table 1:** Univariate demographic characteristics of the analyzed sample (n = 20)

**Variable**	**Point Estimate**	**Standard Deviation**	**Range**
Age, mean years	23.55	3.91	19-34
BMI, mean kg/m^2^	23.84	2.63	19.95-28.68
MPH	2.62	0.55	1.6-3.5
MVPA, min/wk	344	350.52	60-1470
Treadmill attitude (possible range = 1-7)	3.34	1.10	1-5
Gender, % female	55		
Race-ethnicity, %			
Black or African American	15	‏-	‏-
Hispanic	5	‏-	‏-
Asian	15	‏-	‏-
White or Caucasian	65	‏-	‏-
Positive emotions for preferred playlist	2.15	1.53	0.337-7.34
Negative emotions for preferred music playlist	2.56	1.80	0.773-8.99
Positive emotions for imposed music playlist	2.61	0	2.61-2.61
Average motivation score for preferred music playlist (possible range = 7-42)	36.95	5.97	22-42
Average motivation score for imposed music playlist (possible range = 13-130)	78.95	20.06	32-119

Abbreviations: BMI, body mass index; MPH, miles per hour; MVPA, moderate-to-vigorous physical activity.

**Table 2:** Comparison of mean change scores for the Feeling Scale during each visit (n = 20)

**Visit**	**Mean Score**			**Mean Change Score**			
	**Pre**	**Mid**	**Post**	**Pre-to-Mid**	**Mid-to-Post**	**Pre-to-Post** ***P*** ** value**	**Pre-to-Post % Change**
1	3.20	3.55	3.55	0.35*	0	0.35*	10.94
2	3.10	3.35	3.40	0.25	0.05	0.30*	9.68
3	2.85	3.40	3.65	0.55*	0.25	0.80*	28.07
4	3.10	3.65	3.95	0.55*	0.30	0.85*	27.42
5	3.00	3.80	3.75	0.80*	0.05	0.75*	25.00
6	2.75	3.40	3.35	0.75*	0.05	0.60*	21.82
7	2.65	3.65	3.75	1.00*	0.10	1.10*	41.51
8	2.40	3.25	3.35	0.85*	0.10	0.95*	39.58

One-way repeated measures ANOVA for mean change scores between each visit pre-to-mid Feeling Scale was statistically significant along a linear trend (*P* = 0.002). That is, the trend from 0.35 (visit 1) to 0.85 (visit 8) was statistically significant (*P* = 0.002). One-way repeated measures ANOVA for mean change scores between each visit pre-to-post Feeling Scale was statistically significant along a linear trend (*P* = 0.008). That is, the trend from -0.35 (visit 1) to 0.95 (visit 8) was statistically significant (*P* = 0.002). * Statistical significance (*P* < 0.05) when comparing the pre-vs.-post score for each visit in isolation.

**Table 3:** Comparison of mean change scores for the Arousal Scale during each visit (n = 20)

**Visit**	**Mean Score**			**Mean Change Score**			
	**Pre**	**Mid**	**Post**	**Pre-to-Mid**	**Mid-to-Post**	**Pre-to-Post** ***P*** ** value**	**Pre-to-Post % Change**
1	2.60	2.95	2.70	0.35	0.25	*0.10	3.85
2	2.60	2.65	2.60	0.05	0.05	0	0
3	2.70	2.90	3.05	0.20	0.15	0.35	12.96
4	2.40	3.25	3.25	*0.85	0	*0.85	35.42
5	2.50	3.10	3.15	*0.60	0.05	*0.65	26.00
6	2.50	3.05	3.00	*0.50	0.05	*0.45	20.00
7	2.60	3.25	3.40	*0.65	0.15	*0.8	30.77
8	2.20	3.00	2.95	*0.80	0.05	*0.75	34.09

One-Way Repeated Measures ANOVA for mean change scores between each visit pre-to-mid Arousal Scale was statistically significant along a linear trend (*P* = 0.009) and within-subjects (sphericity assumed-*P* = 0.02). That is, the trend from 0.35 (visit 1) to 0.8 (visit 8) was statistically significant (*P* = 0.009). One-Way Repeated Measures ANOVA for mean change scores between each visit pre-to-post Arousal Scale was statistically significant along a linear trend (*P* = 0.05). That is, the trend from 0.1 (visit 1) to 0.75 (visit 8) was statistically significant (*P* = 0.05). * Statistical significance (*P* < 0.05).
